# Layer-dependent nanoscale electrical properties of graphene studied by conductive scanning probe microscopy

**DOI:** 10.1186/1556-276X-6-498

**Published:** 2011-08-18

**Authors:** Shihua Zhao, Yi Lv, Xinju Yang

**Affiliations:** 1 State Key Laboratory of Surface Physics, Fudan University, Shanghai 200433, China

**Keywords:** graphene, scanning capacitance microscopy, electrostatic force microscopy, layer dependence, quantum capacitance

## Abstract

The nanoscale electrical properties of single-layer graphene (SLG), bilayer graphene (BLG) and multilayer graphene (MLG) are studied by scanning capacitance microscopy (SCM) and electrostatic force microscopy (EFM). The quantum capacitance of graphene deduced from SCM results is found to increase with the layer number (*n*) at the sample bias of 0 V but decreases with *n *at -3 V. Furthermore, the quantum capacitance increases very rapidly with the gate voltage for SLG, but this increase is much slowed down when *n *becomes greater. On the other hand, the magnitude of the EFM phase shift with respect to the SiO_2 _substrate increases with *n *at the sample bias of +2 V but decreases with *n *at -2 V. The difference in both quantum capacitance and EFM phase shift is significant between SLG and BLG but becomes much weaker between MLGs with a different *n*. The layer-dependent quantum capacitance behaviors of graphene could be attributed to their layer-dependent electronic structure as well as the layer-varied dependence on gate voltage, while the layer-dependent EFM phase shift is caused by not only the layer-dependent surface potential but also the layer-dependent capacitance derivation.

## 

Graphene is drawing an increasing interest nowadays since its debut in reality [1] as it is a promising material for future nanoelectronic applications [2-4]. While many transport property studies have been carried out by traditional techniques with nanoelectrodes fabricated on graphene [5-8], conductive scanning probe microscopy has recently been applied for direct nanoscale electrical measurements on graphene [9-13]. For example, scanning capacitance microscopy (SCM) was used to study the capacitance of few layer graphene (FLG) [14-16], and the unusual capacitive behavior of graphene due to its quantum capacitance has been found. Electrostatic force microscopy (EFM) was employed to study the electrostatic environment of graphene or to obtain the layer-dependent surface potential of FLG [17,18]. Scanning Kelvin microscopy [19,20] was performed to investigate surface potentials of different graphene layers, and the surface potential was discovered to vary with the layer number. Despite these efforts, the layer-dependent electrical properties, especially the difference between single-layer graphene (SLG) and bilayer graphene (BLG), which is expected to be large due to their different electronic structures, have not been well investigated yet. In this letter, the nanoscale electrical properties of SLG, BLG, and multilayer graphene (MLG with layer number > 2) are investigated by EFM and SCM, and their layer dependences are studied in detail.

The graphene samples were prepared by the mechanical exfoliation method [1] and deposited onto p-type Si substrates coated with a 300 nm of SiO_2 _layer. Although many novel methods have been used to fabricate graphene [21,22], mechanical exfoliation [1] is still a fast and convenient way to obtain high-quality graphene with SLG, BLG, and MLG simultaneously. With the help of optical microscopy to locate the graphene [23], tapping-mode atomic force microscopy (AFM) (MultiMode V, Bruker Nano Surfaces Division, Santa Barbara, CA, USA) has been used to measure the topography. To study the nanoscale electrical properties of graphene, EFM and SCM are performed to investigate the electrostatic force and capacitance behaviors on graphene with different layer numbers. EFM records both the sample topography and the phase shift that is directly linked to the electrical force gradient by using a two-pass method. By SCM, the capacitance variation Δ*C *between the tip and the underlying semiconductor in response to a change in the applied ac bias Δ*V *could be obtained. The detailed operational modes of EFM and SCM have been reported elsewhere [24]. All these experiments were carried out in nitrogen atmosphere at room temperature with Pt-Ir coated Si tips.

Figure 1a shows a typical AFM image of graphene, which contains different graphene layers on SiO_2 _substrate. The profile of the marked line is shown in Figure 1b, which gives the height difference between area A and substrate as well as that between area C and the substrate. The height differences between graphene areas and SiO_2 _substrates are obtained in the same way. As the height of a graphene layer on top of graphene is close to the interlayer distance of graphite [15,25] we fitted the measured graphene height (*h*) as a function of the assigned layer number (*n*) by a straight line: ***h = nt + t*_0_**, as shown in Figure 1c. The fitting result gives the height of a graphene layer *t *= 0.37 nm which is in close agreement with the interlayer distance of graphite (approximately 0.335 nm) and the offset ***t*_0 _= 0.15 nm **which may be caused by the different interaction between tip-graphene and tip-SiO_2 _[15,25]. Thus, the height of SLG is obtained to be **0.37 + 0.15 = 0.52 nm**, which is in agreement with the results of SLG reported in the literatures [14,15]. From the *h*-*n *linear fitting results, area A is termed as SLG, area B as BLG, and area C (four-layer) and D (eight-layer) as MLG.

**Figure 1:**
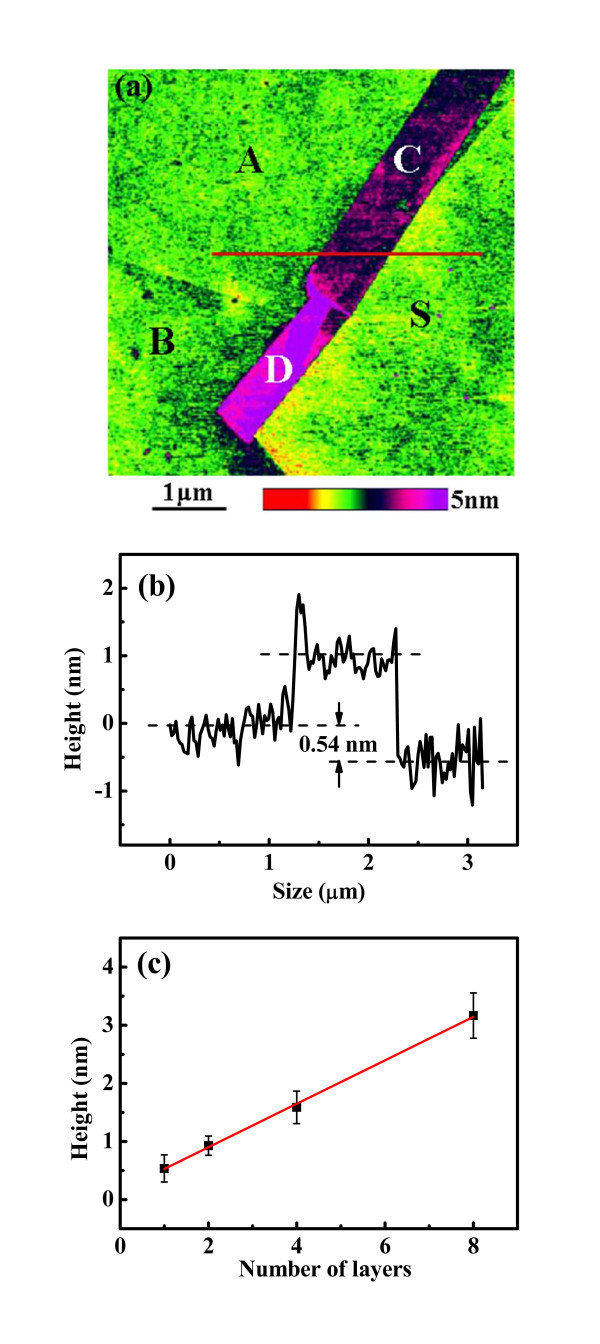
**AFM image of graphene**. **(a) **Tapping-mode height image of the graphene sample. A, B, C, and D are labeled for one-, two-, four-, and eight-layer graphene, respectively, while S is labeled for the SiO_2 _surface. **(b) **The profile of the marked line in (a). **(c) **The measured height (*h*) as a function the assigned number of graphene layers (*n*) and the linear fitting result (red line), giving *h *= 0.37*n *+ 0.15.

SCM measurements were carried out on graphene with different layer numbers, and the images of d*C*/d*V *amplitude at sample DC biases of 0 V and +3 V are shown in Figure 2. The same area is scanned in (a) and (b). The morphology difference of the multilayer rims between (a) and (b) is caused by the coiling of graphene film during the contact-mode scanning. It can be seen that the d*C*/d*V *amplitude does vary with the number of graphene layers, and the differences between SLG, BLG, and MLG can be obviously observed from both images. As the ac voltage variation (Δ*V*) is kept constant in all measurements, the capacitance variation (Δ*C*) obtained by multiplying d*C*/d*V *amplitude with Δ*V *was adopted afterwards instead of the d*C*/d*V *amplitude. The line profiles of Δ*C *obtained on SLG and BLG are shown in Figure 2c, d, respectively. It can be seen that at the DC bias of 0 V, the Δ*C *values of SLG are slightly smaller than those of BLG, but at the DC bias of +3 V, the Δ*C *values of SLG are larger than those of BLG. Figure 2e, f present the averaged Δ*C *with respect to the SiO_2 _substrate for SLG, BLG, and MLG obtained at 0 V and +3 V, respectively. The results show that at the DC bias of 0 V, the Δ*C *measured on graphene increases with *n*. The increase is fast when *n *increases from 1 to 4, and it slows down when *n *increases from 4 to 8. On the other hand, Δ*C *decreases with *n *for the case of +3 V DC bias. Moreover, the Δ*C *values measured on graphene layers are always smaller than those measured on the SiO_2 _substrate for both biases.

**Figure 2:**
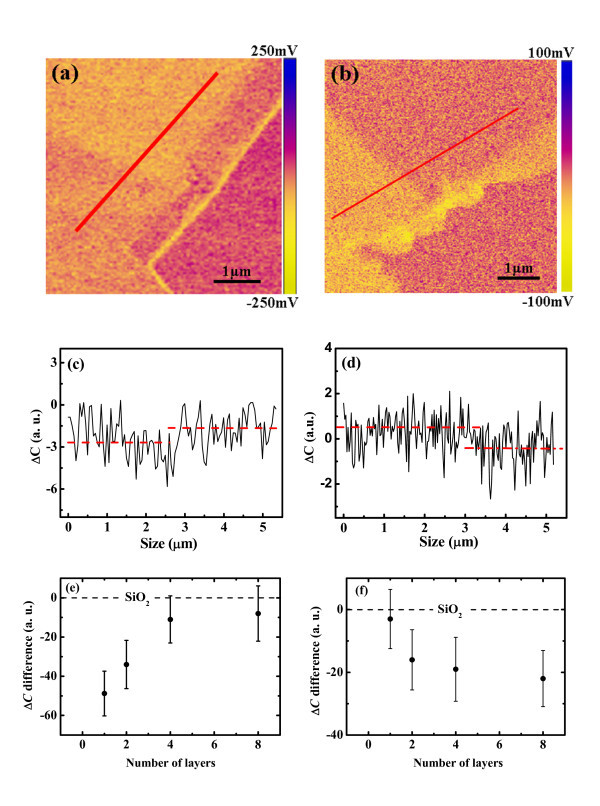
**The dC/dV amplitude images of graphene on SiO_2_**. The dC/dV amplitude images of graphene on SiO_2 _obtained at DC biases of 0 V (a) and +3 V (b). The line profiles of the marked lines (from right top to left bottom) are plotted in (c) and (d) respectively, showing the difference between SLG and BLG. The quantum capacitance variations of graphene with respected to the SiO_2 _substrate as a function of the number of layers at sample DC biases of 0 V and +3 V are shown in (e) and (f) respectively.

As the capacitance measured on graphene is composed of two series capacitance: the quantum capacitance of graphene and the capacitance of the underlying oxide layer, according to the previous studies [14-16], the total capacitance measured on graphene (*C*_tot_) could be written as:

(1) $$C_{\text{tot}}=A_{\text{eff}}{C^{\prime }}_{\text{tot}}=A_{\text{eff}}\frac{{C^{\prime }}_{\text{q}}{C^{\prime }}_{\text{MOS}}}{{C^{\prime }}_{\text{q}}+{C^{\prime }}_{\text{MOS}}},$$

where $A_{e\mathrm{ff}}=\pi {r_{s}}^{2}$ is the effective area of graphene (*r*_s _is the radius of the disk on which the nonstationary electron/hole charge is distributed). *C'*_MOS _and *C'*_q _are the unit area capacitance for tip/SiO_2_/Si structure and graphene, respectively. By considering the contact area, the capacitance measured on SiO_2 _substrate is $C_{MOS}=A_{tip}C_{MOS}^{\prime }$, where$A_{tip}=\pi {r_{tip}}^{2}$ is the tip contact area. Thus, the quantum capacitance *C*_q _can be derived as:

(2) $$C_{\text{q}}=A_{\text{eff}}C_{\text{q}}^{\prime }=\frac{C_{\text{tot}}C_{\text{MOS}}}{C_{\text{MOS}}-\frac{A_{\text{tip}}}{A_{\text{eff}}}C_{\text{tot}}}$$

In Equation 2, *C*_tot _and *C*_MOS _are the capacitances measured on the top of graphene layers and on the SiO_2 _substrate, respectively, but the ratio *A*_tip_/*A*_eff _could not be obtained from the experiments. For FLG, the ratio was found to vary with the gate voltage, as well as the SiO_2 _thickness [14-16]. As reported in the literatures [14], in the case of 300 nm SiO_2 _existed; this ratio for FLG was approximately equal to 1 at the gate voltage of 0 V and changed slightly with the gate voltage, but its relation with *n *is not clear. As a rough approximation, we took ***A*_tip_/*A*_eff _= 1 **for all grephene layers, thus the values of *C*_q _can be calculated from Equation 2. The calculated values for different graphene layers at both DC voltages of 0 and 3 V are shown in Table 1.

**Table 1:** Calculated values for different graphene layers

	**Δ*C***_**q **_**(0 V)**	**Δ*C***_**q **_**(3 V)**	**Increase ratio (Δ*C*_q _(3 V)/Δ*C***_**q **_**(0 V)**
SLG	237	66,453	280
BLG	401	12,096	30
MLG (*n *= 4)	1,521	10,115	7
MLG (*n *= 8)	2,143	8,675	4
*C*_ox_	135	448	-

The calculated quantum capacitance variations of graphene with different number of layers at sample biases of 0 V and +3 V.

From Table 1, it can be seen that at the sample bias of 0 V, the quantum capacitance variation of graphene increases with *n*. With +3 V bias applied, all quantum capacitance variations are much larger than their corresponding values at 0 V. The increase is mostly significant for SLG, which increased about 280 times. The increase magnitude, as shown in Table 1, drops down quickly with increasing *n*. Therefore, the change of graphene quantum capacitance with the DC biases is dependent on *n*, resulting in the different layer-dependent quantum capacitances of graphene at 0 V and +3 V. Since SCM has been performed in the contact mode where the tip contacts with the surface, the DC bias applied between the tip and sample backside acts as the gate voltage. So our results indicate that the capacitance variations increase with the gate voltage for different graphene layers, and the increase magnitude decreases as *n *increases. In previous studies, both the SCM measurements on FLG [14-16] and theoretical studies on SLG [26] showed that the quantum capacitance of graphene increases significantly with the gate voltage. Our results are consistent with those conclusions, but since ***A*_tip_/*A*_eff _= 1 **is used for different graphene layers, it may cause errors for the obtained *C*_q _values, especially at a DC bias of +3 V. Nevertheless, the different quantum capacitance behaviors for graphene with different *n *are definite. As the quantum capacitance represents the density of states (DOS) at Fermi level [26,27] and the DOS of graphene was found to vary with *n *[28], it is reasonable to obtain that the quantum capacitance of graphene is dependent on *n*, as shown in Table 1. On the other hand, it was reported by Yu *et al*. that the work function could be tuned by the gate voltage, where they found that SLG showed larger work function changes with gate voltage than BLG did [18]. They explained the work function change as due to the change in Fermi level (*E*_F_) in graphene, which was different for SLG and BLG. Our results can be interpreted in the similar viewpoint. Different changes of *E*_F _with gate voltage for different graphene layers could result in different carrier density changes with gate voltage, so are the changes of the quantum capacitance with gate voltage.

Meanwhile, the EFM results measured on graphene with different *n *at the sample biases of +2 V and -2 V at a lift height of 20 nm are shown in Figure 3. It is found that for the bias of +2 V, the phase shift difference between SLG and SiO_2 _substrate is smaller than that between BLG and SiO_2_, while for the bias of -2 V, SLG has a larger phase shift with respect to SiO_2 _than BLG. Detailed correlations of the phase shift with *n *obtained at +2 V and -2 V are shown in Figure 3c, d, respectively. The magnitude of the phase shift with respect to the SiO_2 _substrate increases with *n *at +2 V but decreases with *n *at -2 V. In a previous report [18], Datta *et al*. measured the EFM phase shifts on FLG ranged from 2 to 18 layers, and also observed the similar phase shift reverse for two-layer and three-layer graphene at tip biases of -2 V and +3 V. They suggested that the phase shift difference was related with the layer-varied surface potential. This suggestion is doubtful, since the phase shift of electrostatic force is composed of two factors, which could be written as [29]:

**Figure 3:**
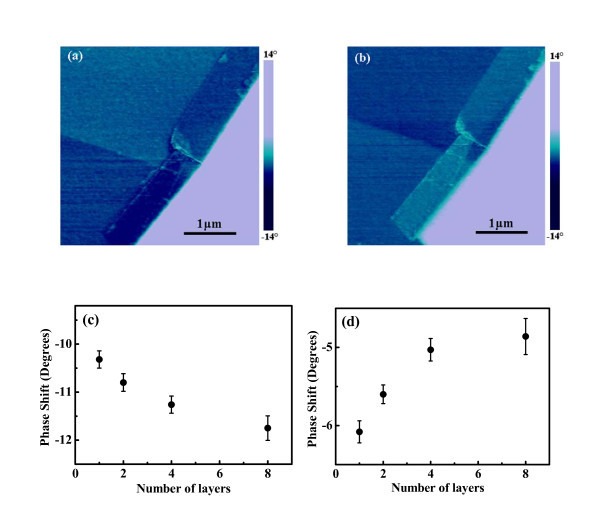
**EFM phase images**. EFM phase images of the same area of Figure 1 at bias voltages of +2 V **(a) **and -2 V **(b)**. The phase shift of graphene with respect to that of SiO_2 _substrate vs the number of graphene layers obtained at +2 V and -2 V are plotted in **(c) **and **(d)**, respectively.

(3) $$\Delta \Phi =-\frac{Q}{k}{grad}_{\text{DC}}F=-\frac{1}{2}\frac{Q}{k}{\left(V_{\text{DC}}-V_{\text{surf}}\right)}^{2}\frac{\partial^{2}C}{\partial z^{2}},$$

where *k *is the stiffness of the cantilever, *Q *is the quality factor, *z *is the tip-sample distance and *C *is the tip-sample capacitance. *V*_DC _is the applied bias, and *V*_surf _is the surface potential correlated with the difference between the tip and sample work functions (***V*_surf _= (*W*_tip_-*W*_sample_)/*e*)**. Hence, both surface potential and capacitance derivation (*∂*^2^*C*/*∂z*^2^) will contribute to the phase shift of electrostatic force. First let's estimate the surface potential contribution (*V*_dc _- *V*_surf_)^2 ^to the different phase shift between SLG and BLG. The work function different between SLG and BLG was reported by Yu *et al*. [20], which is 4.57 eV for SLG and 4.69 eV for BLG, respectively. As the work function of the SiO_2 _substrate is about 5.0 eV and the same tip is applied (PtIr, approximately 4.86 eV), SLG should have a larger phase shift difference with respect to the SiO_2 _substrate than that of BLG for both biases. In other words, the difference in phase shift behavior between SLG and BLG could not only be attributed to their different surface potentials. Thus, the capacitance derivation should be another contribution to the phase shift. Our SCM results aforementioned do indicate that the quantum capacitance of graphene varies with *n*, and it is significantly dependent on the sample biases, which could be expected to induce different EFM phase shifts for different graphene layers at different samples biases.

In conclusion, the nanoscale electrical properties of graphene with different number of layers have been studied by SCM and EFM, and the layer dependences of capacitance variation and EFM phase shift are obtained. SLG, BLG, and MLG exhibit obvious differences in electrostatic force and capacitance behaviors. The different electrical properties obtained on different number of graphene layers could be mainly attributed to their different electronic properties.

## Competing interests

The authors declare that they have no competing interests.

## Authors' contributions

SHZ carried out the experiments. YL participated in the SCM and EFM studies. SHZ and XJY interpreted the results and wrote the manuscript. All authors read and approved the final manuscript.
